# The value of technical characteristics for future performance in youth tennis players: A prospective study

**DOI:** 10.1371/journal.pone.0245435

**Published:** 2021-01-13

**Authors:** Nikki S. Kolman, Barbara C. H. Huijgen, Chris Visscher, Marije T. Elferink-Gemser

**Affiliations:** 1 Center for Human Movement Sciences, University Medical Center Groningen, University of Groningen, Groningen, the Netherlands; 2 Knowledge Center for Sport and Physical Activity, Ede, the Netherlands; 3 Department of Psychology, University of Groningen, Groningen, the Netherlands; Nottingham Trent University, UNITED KINGDOM

## Abstract

The aim of this study is to examine whether technical characteristics predict current and future tennis performance of youth tennis players. Twenty-nine male youth tennis players (age 13.40 ± .51) were assessed on anthropometrical characteristics (height, weight, maturity status) and technical characteristics (ball speed, accuracy and percentage errors) using an on-court tennis test when they were under-14 (U14). Game situations were simulated, which were either fixed or variable. The variable game situations required players to consider the direction of the ball, as opposed to the fixed game situations where players needed to play every ball to the same side. Players’ tennis ratings were obtained U14 (‘current performance’) and under-18 (U18) (‘future performance’). According to their rating U18 players were classified as future elite (n = 9) or future competitive (n = 20). A multiple linear regression analysis showed that ball speed and accuracy were significant predictors of current and future performance (p < .001), with R^2^ of .595 and .463, respectively. When controlling for age, a one-way MANCOVA revealed that future elite players were more accurate than future competitive players (p = .048, 95% CI [.000 to .489]), especially in variable compared to fixed game situations (p < .05). In conclusion, the current study is the first to show that technical characteristics are crucial for current as well as future performance in youth male tennis players. Findings of this prospective study provide essential information to coaches about characteristics that require most attention in performance development in youth players.

## Introduction

Tennis performance results from the interaction of anthropometrical, physiological, psychological, tactical and technical characteristics [1–3]. Maturation, learning and training are the driving forces for the development of these characteristics in youth players. However, the process of performance development is complex and highly varied in talented players [4]. Assessing and monitoring youth tennis performance may help in the successful development of youth players. Knowledge of the possible predictors of current and future tennis performance will provide essential information to coaches about the characteristics that require most attention in their performance development.

Technical characteristics have been demonstrated crucial for tennis performance (for a review see Kolman et al. (2019) [5]). Ball speed and accuracy are usually considered the two most important components of technique in tennis [6]. Professional tennis players are able to direct their strokes both forcefully and accurately to any intended location on the court. An accurate stroke that lacks a high ball speed benefits the opponent, providing this player more time to prepare. Therefore, the combination of speed and accuracy is essential for success in almost every stroke. The number of errors appears critical for tennis match outcome as well. Data analysis of professional tennis tournaments has shown that match winners make fewer unforced errors than match losers [7,8]. Furthermore, research has shown that the number of errors depends on the level of performance. Higher ranked male players make fewer errors than their lower ranked counterparts [5].

Players need to execute their technical characteristics within a tennis-specific situation. They have to adjust their stroke selection according to the tactical situation. In an offensive situation, players have more time to prepare their strokes compared to a defensive situation where they are under time pressure. In addition, technical characteristics depend on players’ ability to anticipate future events. Expert players are faster and more accurate in expecting the direction of their opponents’ strokes than players whose performance levels are lower [5]. Besides being in an offensive situation, outstanding anticipatory skills give players more time to prepare and position themselves. These tactical characteristics determine and limit players’ technical possibilities in a given situation. The reverse is also true as players’ technical characteristics control their tactical possibilities. This means that the tennis-specific situation plays an extremely important role in executing technique in tennis [5]. Still, literature on this topic is scarce.

Earlier research mainly focused on anthropometrical and physical predictors of current tennis performance, unfortunately less is known about predictors of future performance. For example, in a cross-sectional study Ulbricht and colleagues showed that national male players under-14 and under-16 were taller and heavier than their regional counterparts [9]. Height is an advantage in tennis, especially for the serve. Taller players can hit the ball down from a higher point, allowing them to serve at a higher speed than smaller players with the same probability of a successful serve [10,11]. Sprint performance could also benefit from a youth players’ height, because taller players are able to take longer steps [12]. Several physical characteristics, i.e. medicine ball throwing, sprint time, jump height and agility, have also been related to performance. Upper body strength and power were most closely related to tennis performance in youth players [9,13]. Small to moderate correlations were found between these characteristics and ranking (r values ranging from -.17 to -.50) [13]. These results indicate that players with a stronger upper body have a better ranking. Although upper body strength explained 25% of the variance in males’ performance under-13, predicting tennis performance three years later based upon this variable was not possible [13]. These results were not remarkable, because physical fitness of youth players is highly dependent on age and maturity status [14–16]. Youth male tennis players advanced in maturity and age performed better in measures of upper body strength, speed and power [15]. So, older and more mature players have a physical advantage over their relatively younger and less mature opponents. Age and maturation should therefore be taken into consideration when evaluating technical predictors of tennis performance.

The role of technical characteristics in a tennis-specific situation for performance has not been thoroughly investigated yet, especially the role of these characteristics for future performance is unknown. The aim of this prospective study is to examine whether technical characteristics in a tennis-specific situation predict tennis performance under-14 (‘current performance’) and under-18 (‘future performance’) of youth tennis players. Predicting current and future tennis performance with the use of an on-court test, while considering age and maturation, will provide crucial information about performance of youth players in a tennis-specific situation. Findings will contribute to prescribing training programs and monitoring of players’ development. Evaluating crucial characteristics for future performance is necessary for the development of players, as it guides coaches to focus their youth training programs on exercises to improve these characteristics in specific game situations.

## Method

### Participants

Thirty-two male youth players (age 13.4 ± 0.5; body height 167.7 ± 10.6 cm; body mass 52.3 ± 10.9 kg) participated in this study. All players underwent measurements in the pre-season of 2016. Tennis ratings in April 2016 and January 2020 were obtained by using a database of the Royal Dutch Lawn Tennis Association (KNLTB) (www.mijnknltb.nl), when the players were in the age categories under-14 (U14) and under-18 (U18) respectively. In the current study tennis rating U14 is called ‘current performance’, while rating U18 is called ‘future performance’. Three players were excluded from analyses because they stopped with competitive tennis at least one year before January 2020. These three players had similar descriptives (age, height, body mass, maturity status, starting age, tennis training, physical training, ball speed, accuracy, percentage errors and current performance) as the 29 remaining players (P > .05). The remaining players were classified as elite (n = 9) or competitive (n = 20), according to their rating U18. Elite players were those with a rating lower than 3 (range between 1.3 and 2.9), while competitive players were those with a rating higher than 3 (range between 3.0 and 7.8). Players with a rating of 3 or less are among the best 0.6% of all tennis players in the Netherlands. A cut-off value of 3 was chosen to ensure elite players were among the best 1.0% of all tennis players in the Netherlands. This cut-off value makes sense, because players with a rating lower than 3 often do not participate in regional tournaments, but participate in tournaments to earn ranking points in the Netherlands. Informed consent was obtained from players and their parents/legal guardians prior to the measurements after receiving oral and written descriptions of the procedures. The study was approved by the ethical committee of the Medical Faculty of the University of Groningen (Groningen, Netherlands, November 19th, 2015, reference number ECB/2015.11.11_1) and was consistent with the ethical requirements for human experimentation in accordance with the Declaration of Helsinki.

### Procedures

#### Tennis performance

Tennis performance was indicated by players’ individual rating in the Netherlands. The rating represents a player’s general level of play. In the rating system, players are rated on a scale of 9 levels, ranging from 1 to 9 with 4 decimal places. A rating of 9 represents a beginner, while a 1 represents high-level players. The rating is dynamic, which means that a rating is calculated after every match. A player's current rating depends on the results he has achieved and the number of matches played. A result that is achieved in a match depends on the current singles rating of the opponent. When an opponent has a current rating that differs more than 1 point, probably the strongest player wins. If the strongest player actually wins, the result of this match does not count for the determination of the rating. Otherwise a player could receive a worse rating after a win. However, if the weakest player wins, the result does count. The dynamic rating system resembles the International Tennis Number (ITN), a rating system where players are rated on a scale of 10 levels, from ITN 1 to ITN 10.

#### Demographic information

Information on age, age of starting tennis and hours of practice was obtained with a short questionnaire.

#### Maturity status

Testing sessions started with the anthropometrical measurements, which included body height, body mass and sitting height. Players’ body height was measured with a stadiometer and sitting height with a square stool to the nearest millimeter. Body mass was obtained with a digital balance to the nearest 0.1 kilogram. Leg length was calculated by subtracting sitting height from body height [17]. Maturity status was calculated according to the biological age of maturity of each player as described by Mirwald and colleagues [17]. The age at peak height velocity (APHV) is a commonly used indicator of somatic maturity representing the time of the fastest rate of growth in stature during adolescence. This means that a maturity status of -1.0 indicates the player was measured 1 year before this peak velocity; a maturity of 0 indicates that the player was measured at the time of this peak velocity; and a maturity age of +1.0 indicates that the participant was measured 1 year after this peak velocity. Although the method for determining APHV can be inaccurate for early and late maturing boys, it appears to be valid for boys who are on time in maturation and during the period of the growth spurt [18].

#### Dutch Technical-Tactical Tennis Test

**Fig 1:**
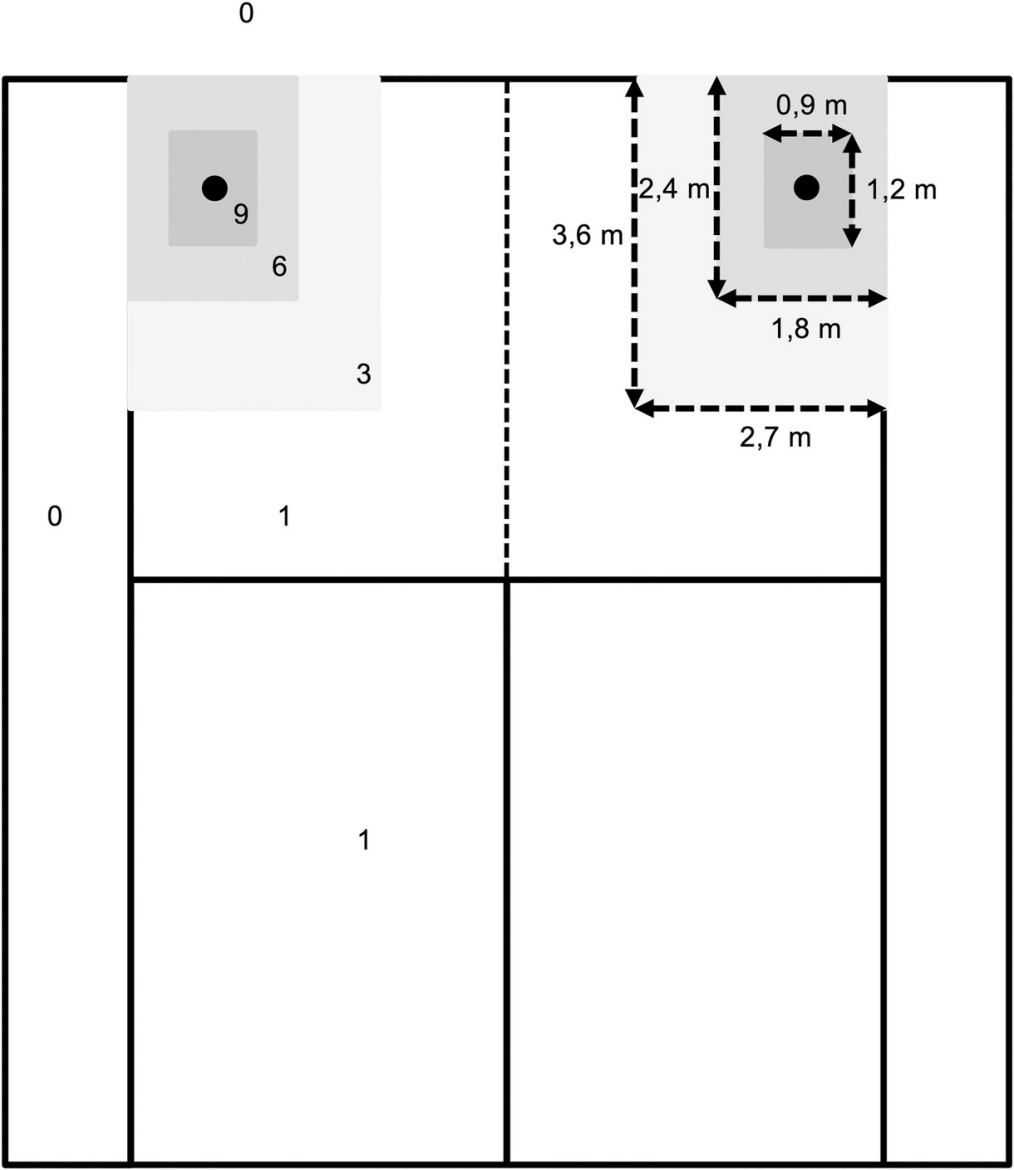
Dimensions of target areas to determine accuracy. This figure demonstrates the half of a tennis court including the dimensions of the target areas and the number of awarded points to balls landing in the areas [19].

Technical characteristics in a tennis-specific situation were assessed with the Dutch Technical-Tactical Tennis Test (D4T) [19]. The D4T simulated games, rallies and various tactical situations (offensive, neutral and defensive situations) with a ball machine (Pro Match Smartshot, Mubo, Gorinchem, the Netherlands) on an indoor tennis court (hardcourt). Before the test, players performed a warm-up of 10 minutes, including 5 minutes of hitting groundstrokes. Players were alternately tested, while the remaining players conducted a training session at low intensity. Measurements took place in the morning or afternoon (10.00 a.m.– 18.00 p.m.), depending on players’ time of training. Ball speed was measured using a radar system (Ball coach pocket radar, PR1000-BC) and ball accuracy was measured using target areas as illustrated in Fig 1.

**Fig 2:**
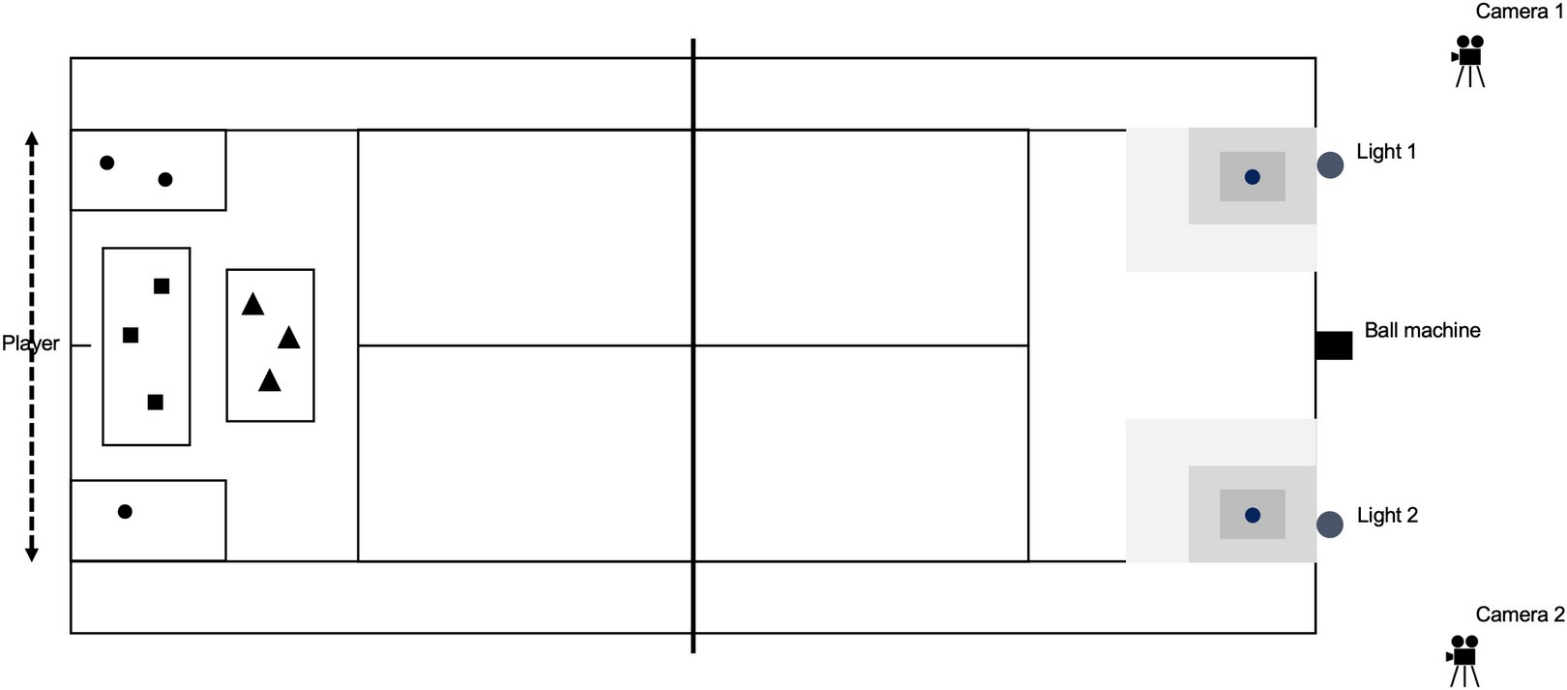
Illustration of the complete test design and various tactical situations. This figure demonstrates an (▲) offensive, (■) neutral and (●) defensive tactical situation. The symbols represent the three ball projections in the tactical situations [19].

The D4T consisted of 72 strokes, grouped in four games of six rallies, in which each rally included three strokes. The various games had an increasing difficulty. In game 1 and game 2, players had to return their strokes to either the left target area (deuce side) or right target area (advantage side). In game 3 players had to alternate their strokes between the left and right target area. In game 4 players had to return their strokes to left or right target area, as indicated by a light that turned red either on the left or right side of the court (Fig 2). The lights were illustrative of the position of an (artificial) opponent. Hence, players had to return their strokes to the opposite side of the red light. The conditions in game 1 and 2 were more fixed compared to the variable conditions in game 3 and game 4 (see Box 1). During the test, players were allowed to rest for 20 seconds in between the rallies and 90 seconds after three games, which was similar to match play.

**Box 1.**

**Table:** 

*An overview of various game situations*	
Game 1 (fixed)	Players have to return their strokes to the left target area (deuce side)
Game 2 (fixed)	Players have to return their strokes to the right target area (advantage side)
Game 3 (variable)	Players have to return their strokes alternately between the left and right target area
Game 4 (variable)	Players have to return their strokes to the opposite side of where the light turned red

Outcome measures included ball speed, accuracy and percentage errors. The D4T has been revealed a reliable and valid instrument to measure technical characteristics in youth players, with an intraclass-correlation coefficient ranging from .73 to .87 and a Spearman’s correlation coefficient of -.75 (P < .001) [19]. Detailed information on the D4T has been reported previously [19].

### Statistical analyses

SPSS Statistics for Windows, version 26 (IBM Corp., Armonk, N.Y., USA) was used for the statistical analyses. Normality of the data was evaluated by exploring normality plots and z-scores for skewness and kurtosis. If values were missing, maximum likelihood estimation was used as substitution method in the missing value analysis. In total 2.1% of the values of the ball speed variable were missing. Relationships between rating U14, rating U18, demographic information, anthropometrical characteristics and technical characteristics were determined by a Pearson’s correlation coefficient. The magnitude of correlation coefficients (r) was considered as small (r = .10), moderate (r = .30) and large (r = .50) [20]. Forward multiple regression analyses were performed using age, starting age, maturity status and technical characteristics, from which a current and future tennis performance (rating) prediction equation was derived. According to the magnitude of correlation coefficients, the magnitude of explained variance (R^2^) was regarded as small (R^2^ = .01), moderate (R^2^ = .09) and large (R^2^ = .25). Detailed analyses were performed to further unravel the importance of technical characteristics for future tennis performance. A one-way multivariate analysis of covariance (MANCOVA) was conducted with rating U18 as grouping factor (elite versus competitive) and accuracy in various games as dependent variables, whilst controlling for age which was considered a covariate. Assumptions for regression analysis and one-way MANCOVA were met. An alpha-level of .05 was used for all significance tests.

## Results

Table 1 shows the descriptive statistics of youth male players at the time of measurements (U14) across future performance level (U18).

**Table 1:** Descriptive statistics of youth male players U14 across performance level U18 (n = 29).

	Elite (n = 9)	Competitive (n = 20)	Total (n = 29)
Age (y)	13.67 ± .45	13.27 ± .51	13.40 ± .51
Height (cm)	175.56 ± 12.28*	165.86 ± 7.53	168.87 ± 10.12
Body mass (kg)	60.90 ± 14.47**	49.74 ± 6.98	53.20 ± 10.98
Maturity status (y)	.55 ± 1.03**	-.44 ± .57	-.14 ± .86
Starting age (y)	4.67 ± 1.32*	5.90 ± 1.29	5.52 ± 1.40
Tennis training (hrs/wk)	10.67 ± 2.65	8.65 ± 3.15	9.28 ± 3.10
Physical training (hrs/wk)	3.44 ± 1.24	2.59 ± 1.19	2.85 ± 1.25
Ball speed (km/h)	104.27 ± 5.08	101.37 ± 7.54	102.27 ± 6.92
Accuracy (pt)	2.59 ± .32**	2.05 ± .52	2.22 ± .52
Percentage errors	34.17 ± 4.84	39.38 ± 9.20	37.76 ± 8.38
Rating U14	4.59 ± .54**	6.47 ± .93	5.88 ± 1.20
Rating U18	2.01 ± .61 **	4.33 ± 1.26	3.61 ± 1.54

*Note*. Data are expressed as mean ± standard deviation

* P < 0.05

**P < 0.01 significantly different from competitive players.

### Relationship among variables

Table 2 presents the correlation coefficients between rating U14, rating U18, (starting) age, height, weight, maturity status and technical characteristics. The values for age, height, weight and technical characteristics represent the values at the initial assessment U14. Results indicated a large positive relationship between rating U14 and rating U18 (r = .91, p < .001). A lower value for rating means a better performance. An inverse relationship was found between rating U14 and other variables such as age, maturity status, ball speed and accuracy (r-values ranging between -.51 and -.65, all p < .001). There was a large positive correlation between rating U14 and percentage errors (r = .51, p < .001). No significant relationship was found between starting age and rating U14 (r = .305, p > .05). Correlations between rating U18 were identical to the variables related to rating U14. These variables were also statistically significant and in the same direction with r-values ranging between -.39 and -.59 (p < .001, p < .05).

**Table 2:** Correlations between rating U14, rating U18, anthropometrical characteristics (U14) and technical characteristics (U14) (n = 29).

	Rating U14	Rating U18	Age	Starting age	Height	Weight	Maturity status	Ball speed	Accuracy	Percentage Errors
Rating U14	1									
Rating U18	.905**	1								
Age	-.516**	-.388*	1							
Starting age	.305	.303	-.135	1						
Height	-.502**	-.430*	.439*	-.055	1					
Weight	-.537**	-.447*	.339	-.100	.926**	1				
Maturity status	-.629**	-.540**	.563**	-.138	.934**	.932**	1			
Ball speed	-.651**	-.589**	.345	-.078	.474**	.513**	.555**	1		
Accuracy	-.639**	-.548**	.442*	-.286	.285	.353	.414*	.401*	1	
Percentage errors	.505**	.458*	-.343	.320	-.232	-.242	-.353	-.234	-.708**	1

*Note*.

* P < 0.05

**P < 0.01.

### Prediction of current and future tennis performance

A multiple linear regression analysis was calculated to predict rating U14 based on age, starting age, maturity status, ball speed, accuracy and percentage errors. A significant regression equation was found for rating U14 (F (2, 26) = 19.085, p < .001), with an R^2^ of .595. Only ball speed and accuracy were statistically significant, with accuracy recording a higher unstandardized beta value (B = -1.038, p = .003, 95% CI [-1.683 to -.393]) than ball speed (B = -.082, p = .002, 95% CI [-.131 to -.033]). Players’ predicted rating U14 (current tennis performance) is equal to 16.575–1.038 (accuracy)—.082 (ball speed), where accuracy is measured in points and ball speed is measured in km·h−1.

A second multiple regression analysis was calculated to predict rating U18 based on the same predictors used for the regression analysis of rating U14. A significant regression equation was found for rating U18 (F (2, 26) = 11.213, p < .001), with an R^2^ of .463. Ball speed and accuracy were again statistically significant, with accuracy recording a higher unstandardized beta value (B = -1.095, p = .025, 95% CI [-2.045 to -.145]) than ball speed (B = -.098, p = .009, 95% CI [-.170 to -.026]). Players’ predicted rating U18 (future tennis performance) is equal to 16.070–1.095 (accuracy)—.098 (ball speed), where accuracy is measured in points and ball speed is measured in km·h−1.

### Accuracy in game situations

**Fig 3:**
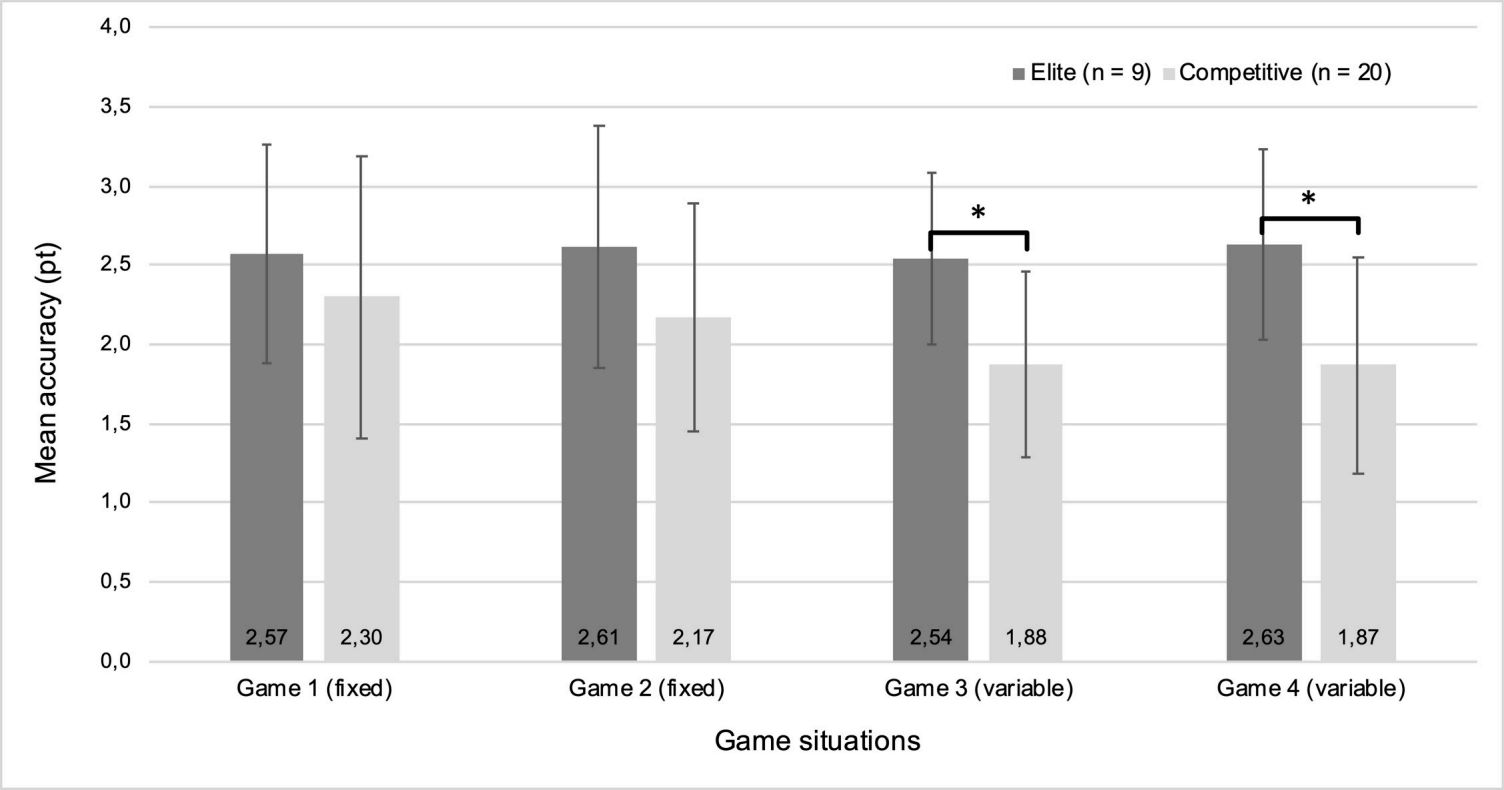
Accuracy in game situations for future elite and competitive players separately. This figure demonstrates the mean accuracy in fixed and variable game situations (errors bars represent standard deviations of the mean). For a detailed overview of various game situations see Box 1; * p < 0.05 in accuracy between future elite and competitive players.

Fig 3 shows the accuracy in every game for future elite and competitive players separately. A one-way MANCOVA was conducted to compare accuracy in various games between future elite and competitive players, whilst controlling for age. There was a statistically significant difference between players’ rating U18 on the combined dependent variables after controlling for age, F (4, 23) = 2.832, p = .048; Wilk's Λ = .670, partial η2 = .330, 95% CI [.000 to .489]. Follow-up analysis showed that future elite players were significantly more accurate than future competitive players in game 3 (p = .038) and game 4 (p = .035), but there were no differences between performance levels in game 1 (p = .606) and game 2 (p = .328) (Fig 3).

## Discussion

To examine whether technical characteristics predict current (U14) and future tennis performance (U18), male youth tennis players were assessed in a tennis-specific situation. A strong relationship was found between various technical characteristics (i.e. ball speed, accuracy and percentage errors) and current as well as future tennis performance. Ball speed and accuracy were found to significantly predict current performance and future performance. Together these predictors accounted for 60% of the variance in current performance and 46% in future performance. These large proportions of explained variance demonstrate that technical characteristics in a tennis-specific situation are extremely important for youth tennis performance.

Anthropometrical characteristics, maturity status and age were associated with performance level. This means that players with a superior rating were taller, heavier, more mature and older. These results deviate from those previously reported with a sample of male players under-12, where no relationship was shown between performance level (ranking) and height, weight, maturity status and age [21]. These players were on average 2.5 years before their APHV, so physical differences between players were probably smaller and less decisive than in the current sample where players were around their APHV. Therefore, the results of the current study were not surprising, because in adolescence biological older players have a physical advantage over smaller and less mature opponents [22]. This was also evident from the higher ball speed that biological older players produced in the current study. However, anthropometrical characteristics, maturity status and age were not able to significantly predict current performance nor future performance. These variables do not seem sensitive enough to predict success in tennis or other racket sports [23]. Considering the significant role of maturation for youth tennis performance [9], predicting future performance based upon these variables seems to be extremely difficult. Current performance may be a sound predictor of future performance, given the strong relationship between current and future performance in the current study (r = .91). However, it provides little insight into the characteristics required for an outstanding tennis performance. In addition, it gives limited information about the components that coaches should focus on to improve performance. It appears therefore crucial to unravel performance characteristics, such as technical characteristics, to prescribe training programs.

Technical characteristics, i.e. ball speed and accuracy, were found to predict current performance U14, which means that youth players who produce fast and accurate balls have a better rating. These results were not remarkable, because hard-hitting players have an advantage over their less hard-hitting opponents, given that an increased ball speed reduces the time for an opponent to return the ball successfully. Similar results have been reported in highly skilled tennis players [6]. Professional players outperformed high-performing youth players for ball speed; however, both groups seem to be able to coordinate various body segments successively, resulting in an efficient groundstroke technique to produce high ball speeds [6,24]. In addition to a higher ball speed, players with a higher performance level demonstrated a higher accuracy in their strokes. The results are in line with earlier research in talented soccer players [25]. To be in control during a match, the combination of ball speed and accuracy is of great importance. According to the speed-accuracy trade-off hypothesis, an increase in the execution time of a movement is required to achieve greater accuracy [26]. In the study on talented soccer players, elite players demonstrated greater accuracy in their ball control, especially under time pressure, compared to sub-elite players [25].

Technical characteristics in a tennis-specific situation also appear extremely important for future performance U18, as indicated by the strong relationship between the various technical characteristics and future tennis performance. It was found that ball speed and accuracy predict future performance of youth tennis players. These results are in line with earlier research in a range of sports, such as field hockey [27], soccer [28] and handball [29]. These studies emphasize the predictive value of technical characteristics for future sports performance. A recent systematic review also demonstrated the great capability of sport-specific technical characteristics assessments to predict future performance [30]. Especially in an early-entry sport as tennis, sport-specific technical characteristics seem to be crucial for future performance given the specific competences to be developed from an early age. Tennis-specific technical characteristics appear to better predict future performance compared to other indicators, such as isolated physical and anthropometrical characteristics that were not found to be significant predictors of future tennis performance [13].

Future elite players were significantly more accurate than future competitive players. In-depth analysis revealed that elite players outperformed competitive players in the variable game situations, but not in the fixed game situations. Elite players were able to maintain their accuracy throughout the game situations, while competitive players became less accurate during the variable game situations. This might be due to the tennis-specificity and increased difficulty of the variable game situations compared to the fixed game situations. The variable game situations required players to consider the direction of the ball, as opposed to the fixed game situations where players needed to play every ball to the same side. In the final (variable) game, players had to look at the other side of the net to see which side the light turned red in order to play the ball to the opposite side. To capture appropriate information, i.e. the side where the light turned red, efficient visual search behaviors were required. These behaviors have been shown crucial for elite tennis players, for example to see an opponents’ actions or the direction of oncoming balls [5]. Executive functions might also have played a role in the current study, given the variable game situations and the information players had to remember where to play the ball (i.e. working memory was required). Elite tennis players might have superior information processing speed, which could have provided them more time to execute their technique properly with the increased demands of the game situations [31].

Despite the fascinating findings of this study, some limitations need to be acknowledged. First, it should be recognized that the D4T measures technical characteristics of groundstrokes in offensive, neutral and defensive rallies, but that it does not capture technique in all game situations. Tennis includes more crucial strokes for performance, like the serve, return and volley strokes. Although the technical characteristics of groundstrokes are crucial for performance, it must be considered that other game characteristics also determine match outcome. Second, it should be acknowledged that predicting future performance U18 is not indicative of becoming a future professional tennis player. Players’ development occurs in a non-linear, unpredictable manner, making it increasingly difficult to predict performance in the distant future (e.g. [4,32,33]). However, gaining knowledge about performance in the near future provides insight into which characteristics require attention in the development of talented players. Third, the results of this study cannot be generalized to other populations without caution. Accordingly, it remains unknown whether the same results apply for female players and other age categories. Fourth, the current study had a relatively small sample size. Although a small number of participants is common in research in high-level competitive sports, caution should be taken in generalizing the findings to professional tennis players. Final, the choice of the Dutch rating system as an indication of tennis performance makes it difficult to compare the findings with other research. However, the Dutch rating system has several advantages compared to the often-used ranking positions. In the dynamic system the rating changes after any match, regardless of whether the match has been played in a tournament or competition. This allow players to better track their progress. Furthermore, the rating system is age neutral and rates all players on the same scale. That makes it easier to compare players of different age categories.

Several practical implications for coaches may be derived from this study. The decisive role of ball speed and accuracy for future performance suggest coaches to focus their youth training programs on exercises to improve these characteristics in variable game situations. For tennis players early in childhood, it seems important to first focus on accuracy. Coordination that is required for accuracy is best developed at a young age [34]. Although coordinative abilities are also required for high ball speed, coaches should focus on this component later in adolescence, because the development of strength (which is important for ball speed) is dependent on the maturity status of players [14]. When players have developed a sufficient degree of accuracy, coaches could focus on gradually increasing the speed of players’ balls. These technical characteristics should be developed in a tennis-specific situation to simulate the context of the match. For future studies it would be interesting to examine whether accuracy in more challenging tennis-specific situations could even better predict future performance, since the distinguishing factor in future performance level is related to accuracy in variable game situations. Technical characteristics accounted for almost half of the variance in future performance; however, a proportion of the variance is still unexplained. Future research should focus on the evaluation of other crucial characteristics including longitudinal assessments to further unravel tennis performance.

In conclusion, the current study was the first to show that technical characteristics in a tennis-specific situation, i.e. ball speed and accuracy, significantly predict current performance U14 as well as future performance U18 in youth male players. Future elite players were more accurate than future competitive players, especially within variable game situations. These findings indicate the relevance of technical characteristics in a sport-specific situation for future performance. By recognizing the importance of ball speed and accuracy in youth players, researchers, coaches and practitioners become more aware of components that require attention in the development of youth tennis players. Knowledge of these predictors contribute to prescribing training programs and monitoring of players’ development.
